# Renoprotective Role and Mechanisms of Luteolin in Chronic Kidney Disease: Insights From NHANES Data, Network Pharmacology, Mendelian Randomization, and Molecular Docking Techniques

**DOI:** 10.1002/fsn3.71236

**Published:** 2025-11-30

**Authors:** Mengjin Li, Kai Hu, Juan Chen, Xinming Li, Cheng Xue, Zhiguo Mao

**Affiliations:** ^1^ Division of Nephrology, Shanghai Changzheng Hospital Naval Medical University Shanghai China; ^2^ Department of Cellular Biology and Anatomy Medical College of Georgia at Augusta University Augusta Georgia USA

**Keywords:** chronic kidney disease, flavonoids, Mendelian randomization, network pharmacology

## Abstract

Although the renoprotective properties of flavonoids are well established in chronic kidney disease (CKD), further investigation is required to identify the most efficacious compound of flavonoids and to elucidate the underlying mechanisms. This study identified the most advantageous compound of flavonoids for CKD using data from the National Health and Nutrition Examination Survey (NHANES) database and investigated underlying mechanisms through mediation analysis, network pharmacology, bioinformatics analysis, Mendelian randomization (MR), and molecular docking techniques. Our research discovered that flavones, including luteolin and apigenin, played a crucial role in reducing the risk of CKD. The intake of apigenin, luteolin, and total flavones was all significantly associated with the risk reduction of CKD. We observed a nonlinear correlation between the luteolin intake and CKD risk, with the optimal daily intake identified as 1.36 mg. Then, we identified luteolin as the most important compound for kidney protection. Mediation effect analysis showed that luteolin might reduce CKD risk by regulating inflammation, serum uric acid, albumin, and bicarbonate. We identified 234 overlapping genes between luteolin and CKD, with enrichment analysis indicating their association with cellular metabolism, inflammation, oxidative stress, and immune responses. Additionally, we identified TP53, HSP90AA1, IL6, and ESR1 as the four hub genes, with HSP90AA1 identified as a risk target for CKD through MR analysis. Molecular docking techniques showed a strong binding affinity between luteolin and HSP90AA1. This study identified luteolin as a key flavonoid linked to lower CKD risk and suggested HSP90AA1 as a potential molecular target mediating its renoprotective effects.

## Graphical abstract

This study identified luteolin as a key flavonoid linked to lower CKD risk. Mediation analysis indicated that lower systemic inflammation, reduced serum uric acid, and higher bicarbonate and albumin levels collectively account for part of this relationship. Network pharmacology highlighted TP53, IL6, HSP90AA1, and ESR1 as central nodes within the luteolin‐associated interactome. MR analysis supported a genetically predicted link between HSP90AA1 and CKD risk, while molecular docking techniques indicated stable binding of luteolin to the HSP90AA1 N‐terminal pocket.

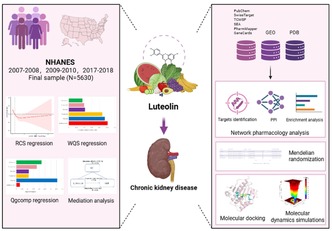

## 1 Background

Chronic kidney disease (CKD) poses a global health challenge, with the prevalence of CKD on the rise in recent decades, resulting in a total of 850 million CKD patients worldwide in 2024 (Francis et al. 2024). CKD imposes a significant disease burden globally, as approximately 1.2 million people died from CKD in 2017, a number that could potentially double to 2.4 million by 2040, or even reach 4 million in worst‐case scenarios (Foreman et al. 2018). The global burden of disease study estimated that CKD was the tenth leading cause of death globally in 2021, and this rank was projected to rise to fifth place by 2050 (GBD 2021 Forecasting Collaborators 2024). CKD progressively impacts overall health, making it essential for patients to adopt lifestyle changes that support better nutrition, regular physical activity, and emotional well‐being. However, due to the complexity of CKD and the wide range of nutrients, both patients and healthcare professionals still lack knowledge about which specific nutrients to prioritize.

Dietary patterns play a significant role in impacting CKD patients, and the beneficial effects of plant‐based diet (PBD) approaches on CKD have been widely acknowledged (Goraya and Wesson 2025; Freeman and Turner 2023). In recent years, many natural products derived from plants have gained popularity due to their diverse biological activities and minimal adverse effects when utilized in disease treatment. Flavonoids, a type of natural polyphenols found in vegetables, fruits, cocoa, oilseeds, and tea, consist of six subclasses: flavanones, flavones, flavonols, flavan‐3‐ols, isoflavones, and anthocyanins (Liu et al. 2023). Several clinical studies have demonstrated the benefits of flavonoids in patients with kidney disease, including anti‐inflammatory action and protective effects on renal function (Borges et al. 2016; Silveira et al. 2020; Yang et al. 2019). A variety of flavonoids have been reported to attenuate DNA damage and modulate inflammation‐related signaling pathways (Alsawaf et al. 2022). Flavonoids such as cyanidin‐3‐glucoside, troxerutin, and luteolin have been shown to exert antioxidant effects through the NRF2 pathway (Rahman et al. 2021; Albarakati et al. 2020). In addition, quercetin, hesperidin, and naringenin have been reported to have antiapoptotic effects (Owumi et al. 2019; Gelen et al. 2021; Elsawy et al. 2021).

Although previous studies have linked dietary flavonoids to better outcomes in CKD, the relative contribution of individual subclasses—and the most relevant single compound—remains ill‐defined. Moreover, the underlying molecular mechanisms are likewise unresolved. This large population‐based study was the first to quantify the effects of each flavonoid subclass on CKD and to identify the compound most strongly linked to CKD outcomes. We used the weighted quantile sum (WQS) regression and the Quantile‐based g‐computation (Qgcomp) regression to identify the most relevant subclasses of flavonoids associated with CKD. Then we utilized weighted logistic regression and Restricted Cubic Spline (RCS) models to elucidate relationships of this leading subclass of flavonoids with CKD risk. To determine which specific compound within the leading subclass was the primary driver, we again applied WQS regression and Qgcomp regression, this time at the individual compound level. The finding of this leading compound prompted us to investigate its mechanisms of action, leading to the mediation and network pharmacology analyses. Finally, Mendelian randomization (MR), molecular docking, and dynamic simulations were integrated to elucidate the potential target through which this leading compound was associated with CKD amelioration. This study will hopefully help to evaluate the therapeutic possibilities of various flavonoids and explore their potential targets, providing a theoretical foundation for the precise and efficient treatment of CKD in the future.

## 2 Materials and Methods

### 2.1 NHANES Database Analysis

#### 2.1.1 Data Sources and Study Population

**FIGURE 1:**
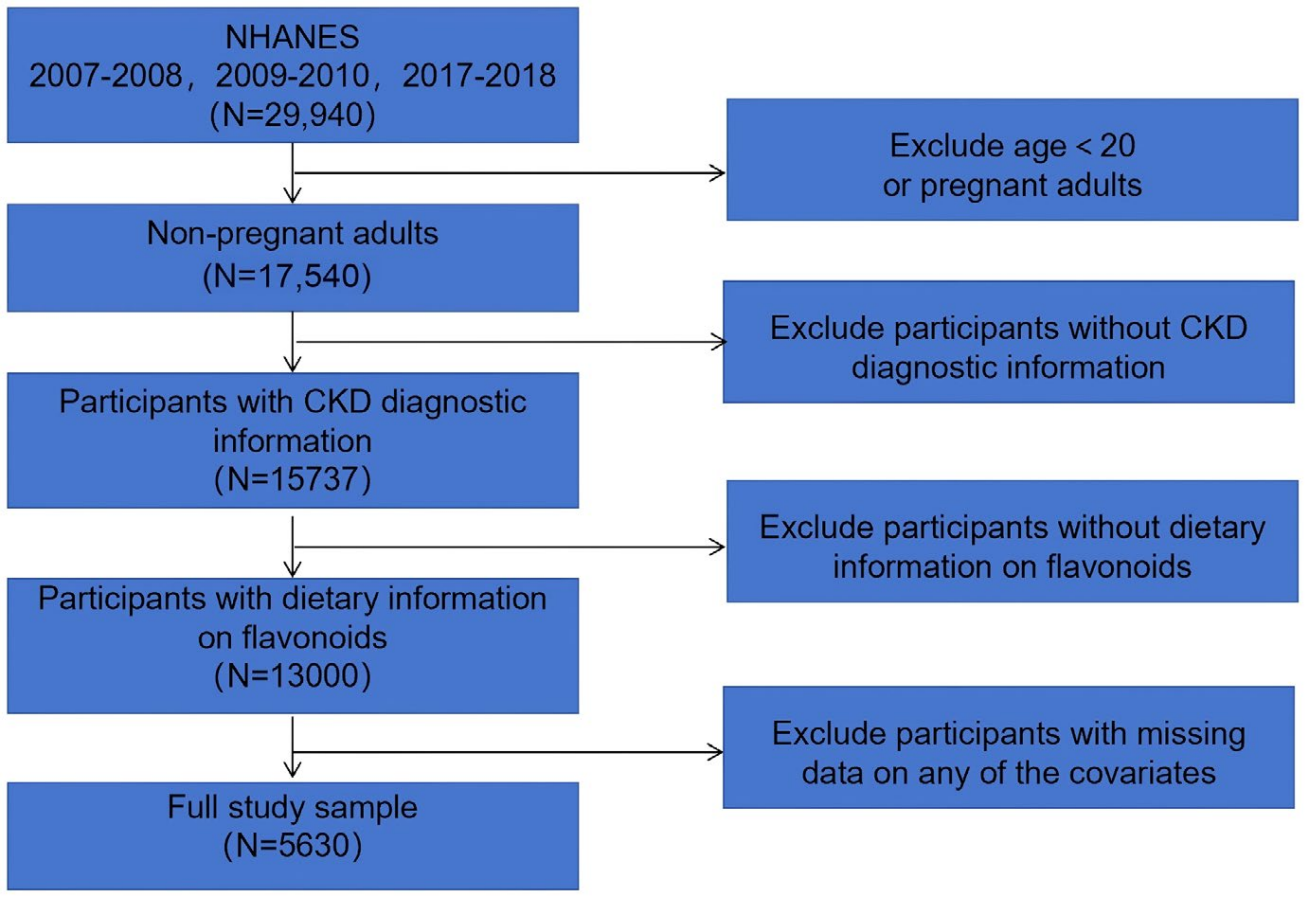
Flowchart of the study.

The National Health and Nutrition Examination Survey (NHANES) is a cross‐sectional survey that aims to gather data on the health and nutritional status of noninstitutionalized Americans. The NHANES interview component includes information on demographics, socioeconomic status, dietary habits, and health‐related issues. For this study, we utilized NHANES data from 2007–2008, 2009–2010, and 2017–2018, all of which were obtained from the official website and are based on public NHANES data. Our selection of these three specific NHANES cycles (2007–2008, 2009–2010, and 2017–2018) was based solely on data availability, for the dietary flavonoid intake data was provided only for these three cycles. Figure 1 provided a detailed illustration of the selection process.

#### 2.1.2 Assessment of Dietary Flavonoid Intake

NHANES researchers collected information from participants about their intake of food and beverages through a 24‐h dietary recall questionnaire. Then, food codes from the USDA Food and Nutrient Database for Dietary Studies (FNDDS) were used to match the various foods and calculate the levels of various nutrients including flavonoids (Fukagawa et al. 2022). We used the average of 2 days of flavonoid intake for this study to better reflect the participants' dietary habits. In this study, total flavonoids referred to the overall content of all flavonoid compounds adjusted by the NHANES study. Dietary data of total flavonoids, six subclasses of flavonoids (including flavanones, flavones, flavonols, flavan‐3‐ols, isoflavones, and anthocyanins) and 29 monomers were included in the NHANES database.

#### 2.1.3 Identification of CKD

In this study, CKD was defined as an estimated glomerular filtration rate (eGFR) < 60 mL/min/1.73 m^2^ and a urinary albumin‐to‐creatinine ratio (uACR) ≥ 30 mg/g. To calculate the eGFR, we utilized the equation provided by the 2009 Chronic Kidney Disease Epidemiology Collaboration (CKD‐EPI).

#### 2.1.4 Covariates

The covariates examined in this study included age, sex (male vs. female), race (Black, White, Mexican, other Hispanic, other race), education level (high school and above vs. less than high school), poverty income ratio (PIR), smoking status (now or former vs. never), physical activity status, diabetes, and hypertension. Physical activity was measured in terms of metabolic equivalent of task (MET) minutes per week. Diabetes was defined as glycated hemoglobin > 6.5%, fasting blood glucose > 7.0 mmol/L, random blood glucose > 11.1 mmol/L, 2‐h oral glucose tolerance test blood glucose > 11.1 mmol/L, use of diabetes mediator or insulin, or a clinical diagnosis of diabetes. Hypertension was defined as SBP > 140 mmHg, DBP > 90 mmHg, use of antihypertensive drugs, or a clinical diagnosis of hypertension.

#### 2.1.5 Statistical Analysis

The percentage of individuals was utilized to characterize the categorical variable, while continuous variables are presented as the mean ± standard deviation. Both the chi‐square test and the independent‐sample *T* test were employed to compare the characteristics of subjects across various groups. We used the WQS regression and the Qgcomp regression to analyze the association between six subclasses of flavonoids and the risk of CKD. The data was divided into a 40% training set and a 60% validation set. The importance of each subcategory was measured according to the weight index. The “flavones” subclass emerged with the highest consistent weight across both models, indicating its strongest association with CKD risk. Given that luteolin and apigenin are the primary constituents of the “flavones” subclass in the NHANES database, we focused on these two compounds. We used weighted logistic regression models to further elucidate the relationships of total flavones, luteolin, and apigenin with CKD risk. Restricted cubic spline (RCS) analysis was used to investigate the nonlinear relationship. Total flavones intake, luteolin intake, and apigenin intake were categorized by quartiles (Q1, Q2, Q3, and Q4). The number of nodes in RCS analysis was set as three (5th, 50th, and 95th). To further determine the leading compound, we repeated the WQS and Qgcomp regression specifically on luteolin and apigenin. Luteolin demonstrated a greater weight than apigenin and was therefore selected for the subsequent mediation analysis to explore potential biological pathways. The proportion mediated (%) was defined as the ratio of the mediating effect to the total effect, serving as an indicator of the effectiveness of the mediators.

The data were analyzed using R version 4.3.3. The R package “survey” was used to weight the data. WQS regression was performed using the R package “gWQS,” while Qgcomp regression was carried out using the R package “qgcomp.” RCS analysis was conducted using the R package “rms.” Moreover, mediation analysis was performed using the R package “mediation.” *p* < 0.05 was considered to be significant.

### 2.2 Network Pharmacology

#### 2.2.1 Targets Identification

The structure of luteolin was retrieved from PubChem (https://pubchem.ncbi.nlm.nih.gov/). Putative pharmacological targets were predicted with SwissTargetPrediction (http://swisstargetprediction.ch/), TCMSP (https://www.tcmsp‐e.com/), SEA (https://sea.bkslab.org/), and PharmMapper (https://www.lilab‐ecust.cn/pharmmapper). Non‐human proteins were mapped to their human orthologs where applicable. CKD‐related genes were downloaded from GeneCards (https://www.genecards.org/); genes with a relevance score > 10 were retained. Overlapping targets between luteolin and CKD were identified with a Venn diagram.

#### 2.2.2 Protein–Protein Interaction (PPI) Networks Construction and Hub Genes Identification

We entered the above targets into the String database (http://www.string‐db.org/), restricted the species to “ *Homo sapiens*,” set the “Minimum required interaction score” to “High Confidence (0.700),” and selected the “FDR Stringency Value” as “High.” Cytoscape (version 3.9.1) was used to obtain core genes.

#### 2.2.3 Enrichment Analysis

We performed Kyoto Encyclopedia of Genes and Genomes (KEGG) analysis and Gene Ontology (GO) analyses using the R package “clusterProfiler,” and *p* < 0.05 was considered to be significantly enriched.

#### 2.2.4 Hub Genes' Single‐Gene Gene Set Enrichment Analysis (GSEA)

We obtained the CKD‐related dataset GSE66494 (Agilent‐014850 Whole Human Genome Microarray 4x44K G4112F) through the Gene Expression Omnibus (GEO) database (http://www.ncbi.nlm.nih.gov/geo). We first ordered the gene set by the Pearson correlation between the expression of all genes and the expression of target genes, and then conducted the single‐gene GESA by using the R package “fgesa.”

#### 2.2.5 Analysis of the Level of Immune Cell Infiltration and the Correlation Between the Hub Gene and the Level of Immune Cell Infiltration

We detected the presence of immune cells in both the normal and CKD groups within the GSE66494 dataset using the R package “xCell.” Subsequently, we computed the Pearson correlation between immune cells and hub genes. *p* < 0.05 was considered statistically significant.

### 2.3 Mendelian Randomization Analysis

We performed an Expression Quantitative Trait Locus (eQTL) analysis on the hub genes associated with CKD, using SNPs from European GWAS datasets (GWAS IDs were available in the Methods in Supporting Information) and the FinnGen dataset for CKD (GWAS ID: finn‐b‐N14_CHRONKIDNEYDIS). The inverse variance weighted (IVW), MR‐Egger, weighted median, weighted mode, and simple mode were conducted to evaluate the results, with a *p* value threshold for eQTL set at 5e‐08. The primary analysis method was IVW, which was suitable for most scenarios. Results calculated by the other methods were shown in Table S1. We conducted heterogeneity tests by using IVW and MR‐Egger (Table S2), while MR‐Egger was used to assess horizontal pleiotropy (Table S3). The “TwoSampleMR” package was utilized for MR analysis. *p* < 0.05 was considered statistically significant.

### 2.4 Molecular Docking

The 3D structure files of the proteins were downloaded from the RCSB PDB database, ID: 1BYQ (https://www.rcsb.org/structure/1BYQ). Docking analysis of the protein structures was performed using PyMOL 2.5.4 and Discovery Studio Visualizer (BIOVA) software. The 3D structure files of luteolin were obtained from the PubChem database and optimized by the MMFF94 force field of OpenBabel 3.1.1 software to obtain the best molecular structure at the lowest energy state. Autodock Vina‐based docking was realized using CB‐Dock2 (Liu, Yang, et al. 2022).

### 2.5 Molecular Dynamics Simulations

Molecular dynamics simulations were performed using Gromacs2020.6 software. We analyzed the molecular dynamics simulation trajectories, including root mean square deviation (RMSD), root mean square fluctuation (RMSF), radius of gyration (Rg), solvent‐accessible surface area (SASA), and the relative free energy distributions. Specific descriptions can be found in the Methods in Supporting Information.

## 3 Results

### 3.1 Characteristics of Included Participants From NHANES Database

Table 1 summarized the baseline characteristics of the 5630 participants, 1039 (18.5%) of whom had CKD. Compared with non‐CKD individuals, those with CKD were significantly older, more likely to be non‐Hispanic White, had lower educational attainment and income, were less physically active, and exhibited higher prevalences of hypertension and diabetes (all *p* < 0.05). Total flavonoid intake was markedly lower in the CKD group, and significant between‐group differences were also observed for the four major subclasses: flavonols, flavones, anthocyanidins, and isoflavones.

**TABLE 1:** Characteristics of study.

Characteristics	CKD patients (*N* = 1039)	Non‐CKD patients (*N* = 4591)	*p*
Age (years)	64.5 ± 15.3	47.7 ± 16.4	< 0.001
Sex (*n*)			
Female	563 (54.2%)	2376 (51.8%)	0.167
Male	476 (45.8%)	2215 (48.2%)	
Race (*n*)			
Mexican American	141 (13.6%)	764 (16.6%)	< 0.001
Non‐Hispanic Black	206 (19.8%)	866 (18.9%)	
Non‐Hispanic White	553 (53.2%)	2119 (46.2%)	
Other Hispanic	76 (7.3%)	462 (10.1%)	
Other Race	63 (6.1%)	380 (8.3%)	
Education level (*n*)			
High school or above	717 (69%)	3546 (77.2%)	< 0.001
Less than high school	322 (31%)	1045 (22.8%)	
PIR	2.4 ± 1.5	2.6 ± 1.6	< 0.001
Smoking status (*n*)			
Never	547 (52.6%)	2533 (55.2%)	0.149
Now or former	492 (47.4%)	2058 (44.8%)	
MET per week	46.7 ± 14.0	43.2 ± 12.1	< 0.001
Hypertension (*n*)			
No	268 (25.8%)	2905 (63.3%)	< 0.001
Yes	771 (74.2%)	1686 (36.7%)	
Diabetes (*n*)			
No	543 (52.3%)	3840 (83.6%)	< 0.001
Yes	496 (47.7%)	751 (16.4%)	
Total sum of all 29 flavonoids (mg)	178.7 ± 314.6	200.9 ± 341.3	0.044
Total flavonols (mg)	15.2 ± 14.8	17.7 ± 16.4	< 0.001
Total flavones (mg)	0.7 ± 0.9	0.9 ± 1.7	< 0.001
Total flavanones (mg)	12.5 ± 22.2	13.9 ± 27.1	0.081
Total flavan‐3‐ols (mg)	138.6 ± 301.0	153.8 ± 324.6	0.149
Total anthocyanidins (mg)	10.7 ± 21.7	12.7 ± 29.2	0.011
Total isoflavones (mg)	1.0 ± 5.7	1.9 ± 10.3	< 0.001

*Note:* Data were presented as weighted percentages or means ± standard deviation.

Abbreviations: CKD, chronic kidney disease; MET, metabolic equivalent of tasks; PIR, poverty income ratio.

### 3.2 The Association Between Flavonoid Mixture Intake and CKD

**FIGURE 2:**
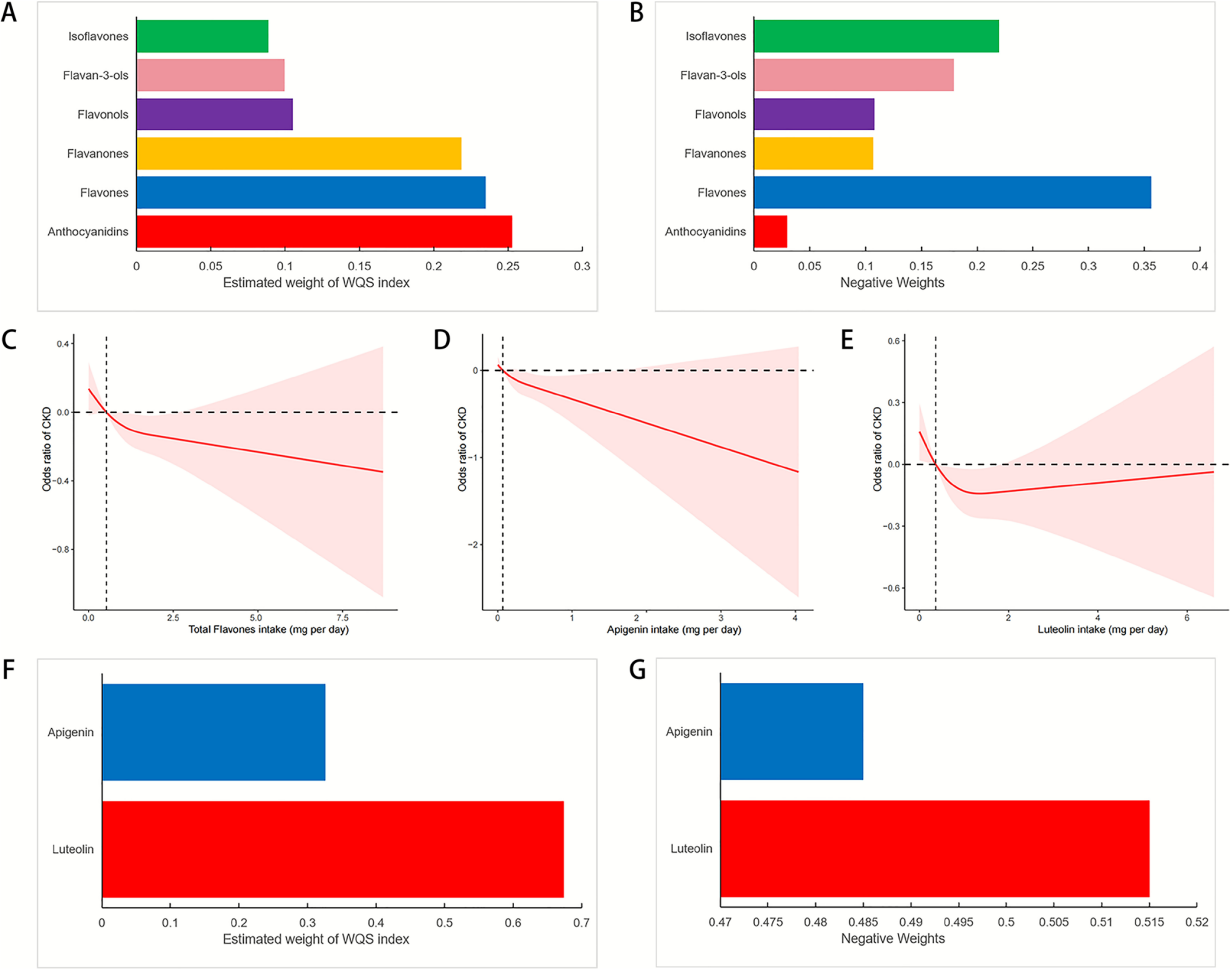
Results from the WQS regression, the Qgcomp regression, and the RCS analysis. (A) WQS model regression index weights for flavonoid subclasses. (B) Qgcomp model regression index weights for flavonoid subclasses. (C) The dose–response relationship between the total flavones intake and the odds ratios of CKD. (D) The dose–response relationship between the apigenin intake and the odds ratios of CKD. (E) The dose–response relationship between the luteolin intake and the odds ratios of CKD. (F) WQS model regression index weights for luteolin and apigenin. (G) Qgcomp model regression index weights for luteolin and apigenin. All models were adjusted for sex, age, race, education, PIR, MET per week, smoking status, hypertension, and diabetes.

We applied WQS and Qgcomp regression to estimate the combined association of six flavonoid subclasses with CKD. Both models treated CKD status as the outcome and intake of six subclasses of flavonoids as the exposure, adjusting for age, sex, race, education, income, BMI, smoking, physical activity, hypertension, and diabetes. The WQS index was inversely associated with CKD (OR 0.849; 95% CI 0.733–0.984; *p* = 0.030). Anthocyanidins contributed 25.3% of this weighted signal, followed by flavones (23.5%) and flavanones (21.9%) (Figure 2A). Qgcomp yielded a similar inverse association (OR 0.781; 95% CI 0.678–0.898; *p* < 0.001), with flavones accounting for 35.6% of the mixture effect (Figure 2B). Because flavones carried significant weight in both approaches, they emerged as the subclass most consistently related to lower CKD prevalence.

### 3.3 The Association Between Flavone Intake and CKD

Table 2 demonstrated the relationship between consumption of total flavones, luteolin, and apigenin to CKD. As compared to the lowest quartile (Q1), the unadjusted logistic regression model showed no significant association between CKD and total flavonoid intake, luteolin intake, or apigenin intake. After adjusting for age, sex, and race, inverse associations could be found between intake of total flavonoids (Q3, Q4), luteolin (Q3, Q4) as well as apigenin (Q4) and the prevalence of CKD. However, only luteolin (Q3) remained significantly associated with lower CKD prevalence in the fully adjusted model.

**TABLE 2:** Association between flavones intake and CKD adjusted by weighted logistic regression.

	OR (95%CI)		
	Model 1	Model 2	Model 3
Total flavones (mg per day)			
Q1 [0, 0.185]	ref	ref	ref
Q2 (0.185, 0.525]	0.95 (0.76, 1.20)	0.87 (0.66, 1.13)	0.88 (0.66, 1.18)
Q3 (0.525, 1.11]	0.83 (0.64, 1.07)	**0.71 (0.52, 0.96)**	0.79 (0.57, 1.10)
Q4 (1.11, 87.245]	0.89 (0.69, 1.16)	**0.72 (0.53, 0.97)**	0.83 (0.61, 1.13)
Apigenin (mg per day)			
Q1 [0, 0.015]	ref	ref	ref
Q2 (0.015, 0.07]	1.02 (0.73, 1.42)	1.03 (0.73, 1.47)	1.08 (0.75, 1.56)
Q3 (0.07, 0.215]	1.01 (0.73, 1.38)	0.94 (0.68, 1.29)	0.98 (0.69, 1.38)
Q4 (0.215, 68.61]	0.87 (0.65, 1.17)	**0.72 (0.52, 0.99)**	0.80 (0.55, 1.15)
Luteolin (mg per day)			
Q1 [0, 0.12]	ref	ref	ref
Q2 (0.12, 0.365]	1.08 (0.86, 1.36)	0.97 (0.75, 1.26)	1.00 (0.76, 1.30)
Q3 (0.365, 0.865]	0.78 (0.59, 1.03)	**0.65 (0.47, 0.91)**	**0.72 (0.52, 1.00)**
Q4 (0.865, 18.635]	0.89 (0.67, 1.17)	**0.72 (0.53, 0.96)**	0.84 (0.62, 1.14)

*Note:* Bolded ORs were statistically significant. Model 1 did not include any covariate. Model 2 was adjusted for age, race and sex. Model 3 was adjusted for sex, age, race, education, PIR, MET per week, smoking status, hypertension and diabetes.

Abbreviations: CI, confidence interval; OR, odds ratio; Q1, quartile 1; Q2, quartile 2; Q3, quartile 3; Q4, quartile 4.

RCS analyses were fitted to evaluate dose–response patterns. Figure 2C–E showed approximately linear inverse trends for total flavones and apigenin across the observed range, whereas for luteolin, the curve plateaued at intakes ≥ 1.36 mg/day.

Finally, we calculated the relationship between the flavones mixture and the prevalence of CKD. Both WQS (OR 0.853; 95% CI 0.768–0.947; *p* < 0.01) and Qgcomp (OR 0.837; 95% CI 0.770–0.911; *p* < 0.01) indicated that higher combined flavones intake was associated with lower CKD prevalence, with luteolin contributing the largest share of the weighted signal (Figure 2F,G).

### 3.4 Mediation Analysis Between Luteolin Intake and CKD

**FIGURE 3:**
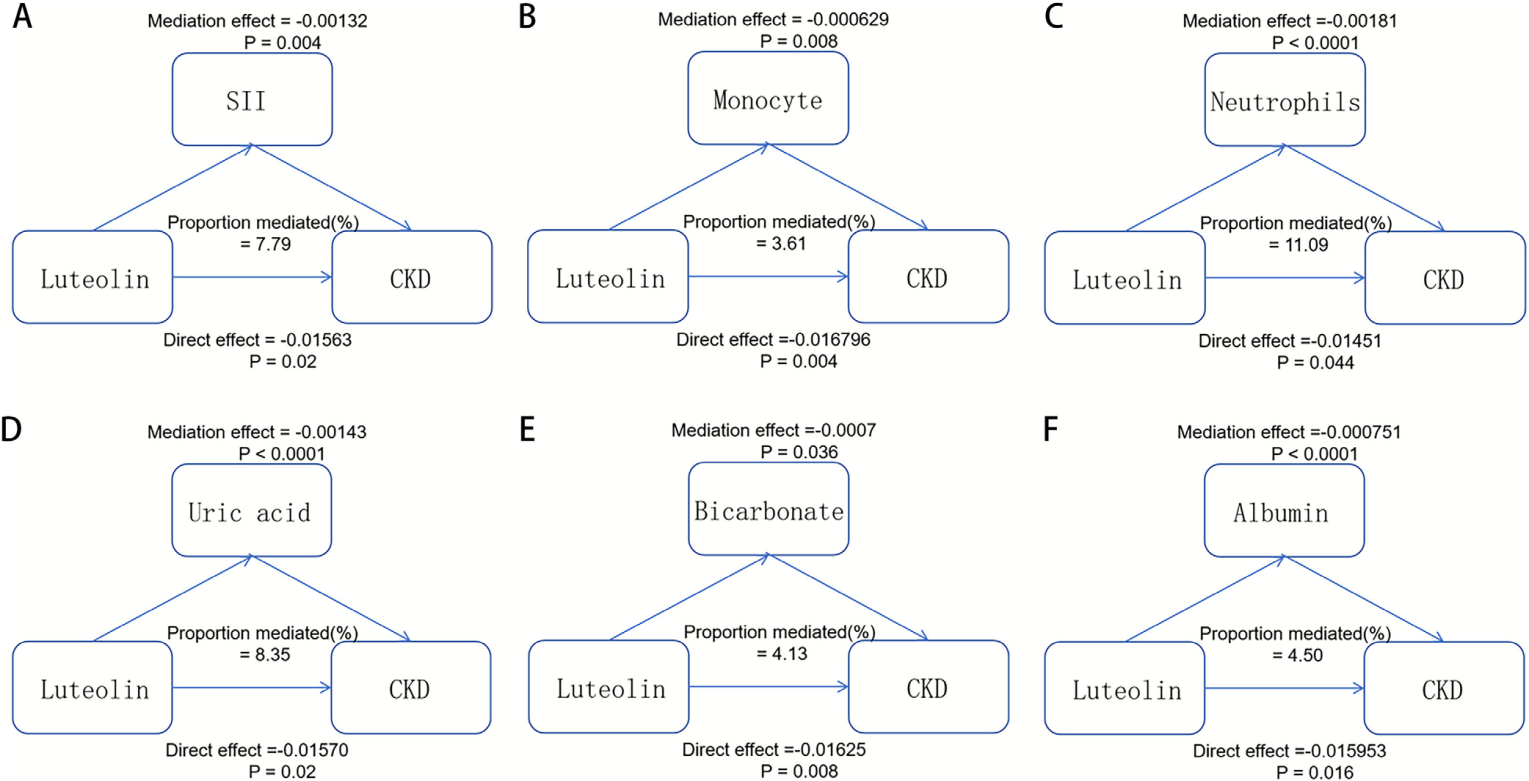
Estimated proportion of the association between luteolin intake and CKD mediated by various factors. (A) SII, systemic immune‐inflammation index. (B) Monocyte. (C) Neutrophils. (D) Uric acid. (E) Bicarbonate. (F) Albumin. All models were adjusted for sex, age, race, education, PIR, MET per week, smoking status, hypertension, and diabetes.

Systemic immune‐inflammation index (SII) is a holistic inflammatory marker that integrates the levels of neutrophils, platelets, and lymphocytes in peripheral blood to evaluate the overall inflammatory and immune status of the body. The mediating effect of SII accounted for 7.99% of the total effect, which was mainly mediated through neutrophils (proportion mediated = 11.09%) and monocytes (proportion mediated = 3.61%) (Figure 3A–C). Besides that, serum uric acid (proportion mediated = 8.35%), albumin (proportion mediated = 4.5%), and bicarbonate (proportion mediated = 4.13%) were also mediators we found between the CKD risk reduction and the luteolin intake (Figure 3D–F).

### 3.5 Network Pharmacology

**FIGURE 4:**
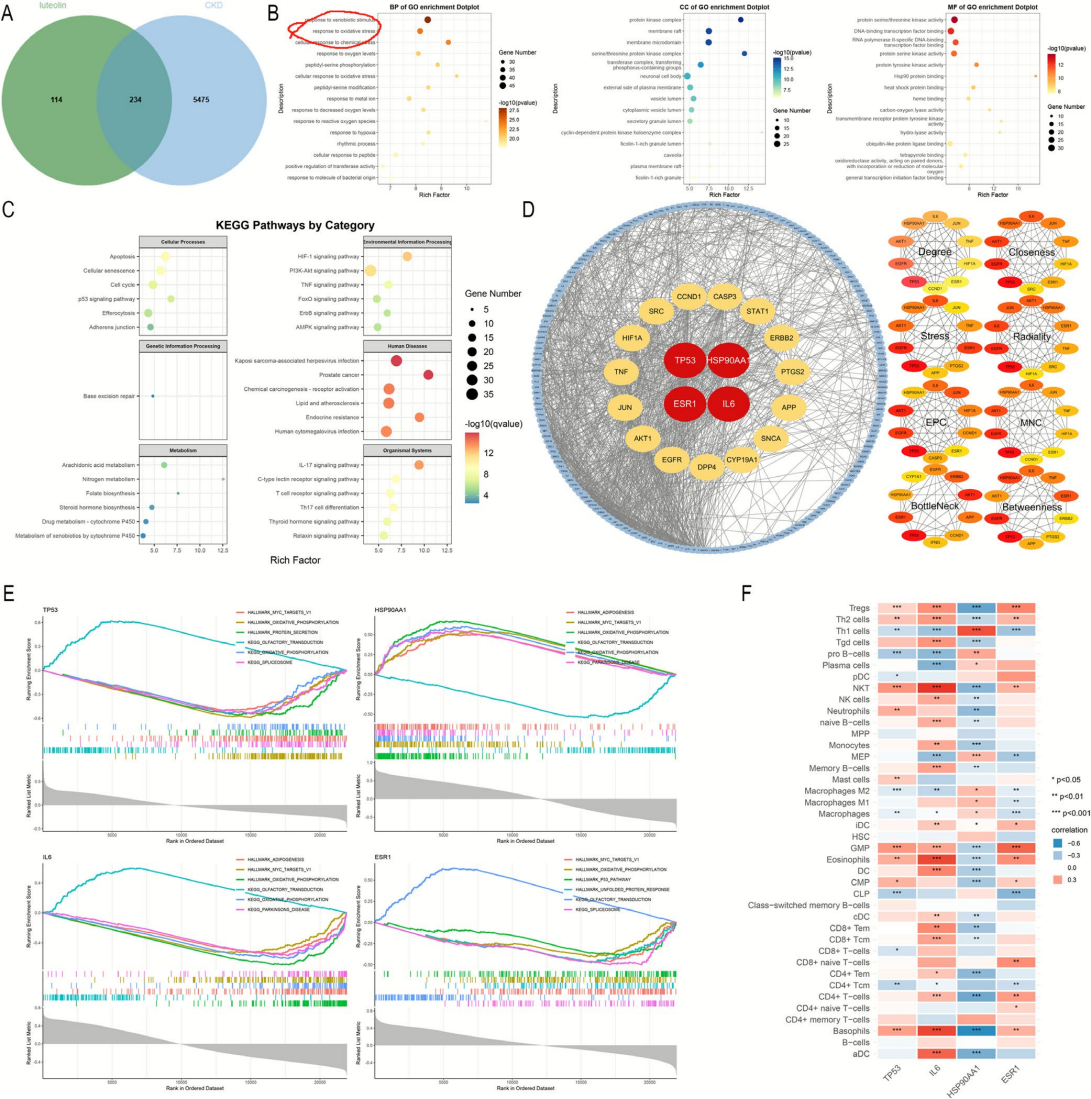
Identification of luteolin‐related CKD targets, construction of PPI network, enrichment analysis, and immune‐infiltration analysis. (A) Identification of potential targets of luteolin in CKD. (B, C) GO and KEGG enrichment analysis of identified potential targets of luteolin in CKD. (D) PPI network of potential targets of luteolin in CKD. (E) Single‐gene GSEA of four hub genes. (F) The correlation between hub genes' expression and immune cell infiltration.

We identified 234 overlapping targets (Materials 1 in Supporting Information) by aligning 348 luteolin‐related action genes with 5709 CKD‐related targets (Figure 4A). Subsequently, we conducted enrichment analysis to explore the potential biological functions of these target genes. GO analysis revealed that these core genes were primarily associated with stimulus response, oxidative stress regulation, chemokine regulation, apoptosis, and cell‐cycle regulation (Figure 4B). KEGG analysis showed the related pathways, including HIF‐1, PI3K‐AKT, IL‐17, and TNF (Figure 4C). These findings suggested that cellular metabolism, oxidative stress, inflammation, and immune response might play important roles in the beneficial effects of luteolin on CKD.

We created a PPI network for 234 overlapping genes and identified them using eight different algorithms (Degree, Closeness, Stress, Radiality, EPC, MNC, BottleNeck, and Betweenness) in Cytoscape software (Figure 4D). Eventually, we identified four hub genes: Tumor Protein 53 (TP53), Heat Shock Protein 90 Alpha Family Class A Member 1 (HSP90AA1), Interleukin 6 (IL6), and Estrogen Receptor 1 (ESR1).

### 3.6 GSEA Analysis

To further explore the potential mechanisms of luteolin, we performed single‐gene GSEA enrichment analysis of four hub genes using the GSE66494 dataset. The findings revealed a strong correlation between the expression of these hub genes and various biological pathways, including “oxidative phosphorylation” and “Myc targets” (Figure 4E).

### 3.7 Immune Cell Infiltration and Correlation of Hub Genes With Immune Cells

Compared with normal kidneys, CKD tissues exhibited markedly altered immune cell infiltration patterns. We found a significant association between hub genes, especially IL6 and HSP90AA1, with immune cell infiltration. IL6 was found to be in a positive correlation with the presence of natural killer (NK) cells, dendritic cells (DC), T helper type 2 (Th2) cells, and regulatory T cells, as well as in a negative correlation with T helper type 1 (Th1) cells (Figure 4F). Interestingly, the relationship of HSP90AA1 with these cell types was opposite to that of IL6. Additionally, the expression of HSP90AA1 was positively linked to the infiltration of macrophages (Figure 4F). This indicated that luteolin might impact the progression of CKD by regulating the extent of immune cell infiltration.

### 3.8 MR Analysis

**FIGURE 5:**
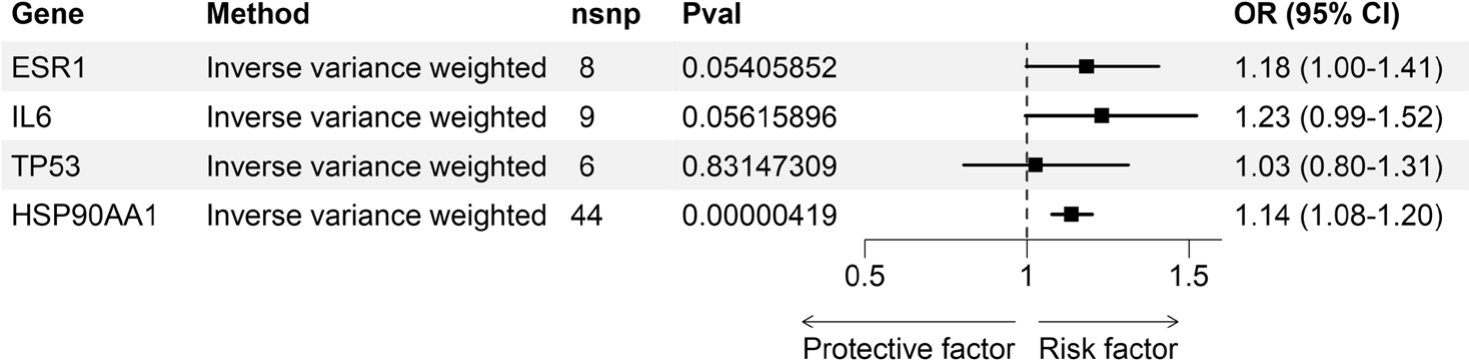
Results from MR analysis of overlapping genes and CKD.

Figure 5 showed that HSP90AA1, identified as a high‐risk target, exhibited a significant relationship with CKD (OR: 1.137, 95% CI: 1.077–1.201, *p* < 0.01). ESR1 (OR: 1.184, 95% CI: 1.000–1.405, *p* = 0.054), IL6 (OR: 1.231, 95% CI: 0.995–1.523, *p* = 0.056), and TP53 (OR: 1.026, 95% CI: 0.804–1.311, *p* = 0.831) were all risk factors for CKD, but not statistically significant. No horizontal pleiotropy or heterogeneity was detected (Tables S2 and S3). The *F*‐statistics for all instrumental SNPs far exceed the conventional threshold of 10 (Table S4).

### 3.9 Molecular Docking and Molecular Dynamics Simulations

**FIGURE 6:**
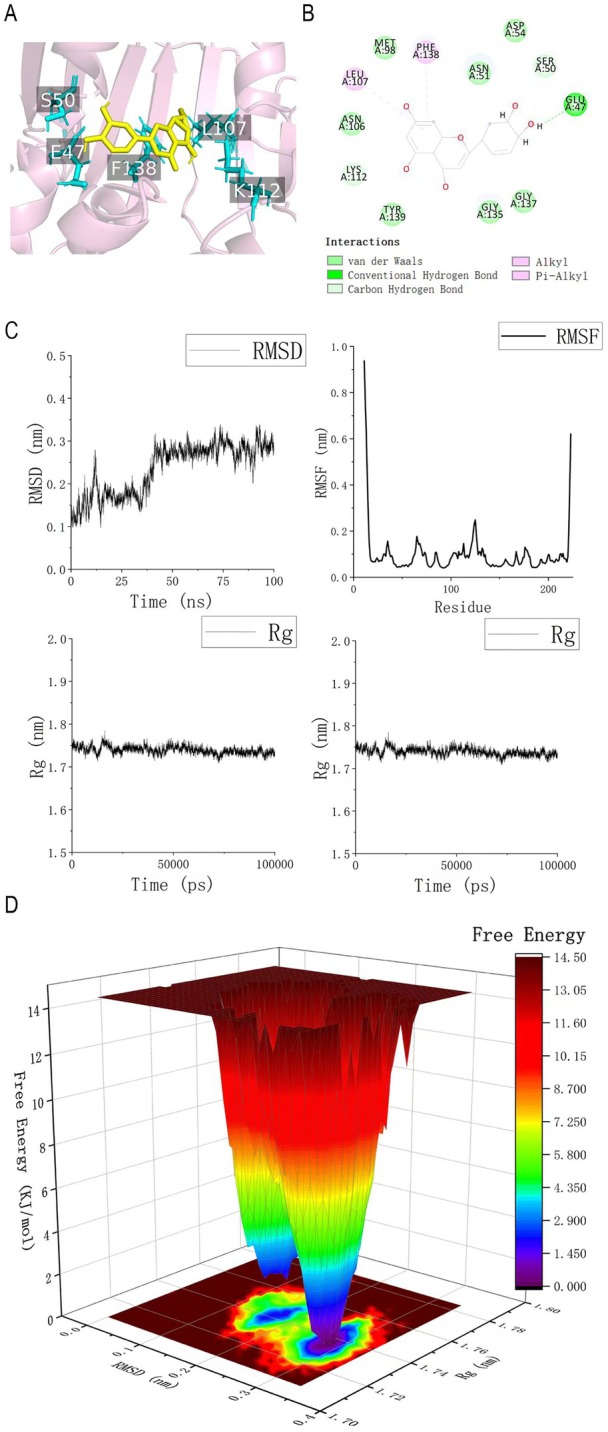
Results of leuteolin‐HSP90AA1 system's molecular docking and molecular dynamics simulation. (A, B) Protein–ligand interactions of the leuteolin‐HSP90AA1 system. (C) RMSD, Rg, SASA, and RMSF of HSP90AA1 in the molecular dynamics simulation. (D) Free energy landscape of HSP90AA1 in the molecular dynamics simulation.

The binding energy between luteolin and HSP90AA1 was −7.4 kcal/mol, indicating good binding between them. The conformations corresponding to the lowest free energy during the molecular dynamics simulation are shown in Figure 6A,B. Residue Glu47 of HSP90AA1 forms one conventional hydrogen bond and two C–H bonds with luteolin; Ser50 forms three C–H bonds with luteolin; Lys112 forms one C–H bond with luteolin; Leu107 forms one alkyl bond with luteolin, and Phe138 forms one pi‐alkyl bond with luteolin. These residues, particularly Glu47 and Ser50, are located within the N‐terminal ATP‐binding domain of HSP90AA1, suggesting that luteolin may interfere with its ATPase activity and chaperone function. As shown in Figure 6C, the RMSD curve recovered to be stable after 40 ns, and the range of curve fluctuation was around 0.5 nm, suggesting the stable binding of luteolin to the HSP90AA1 protein. Both the Rg and SASA of the complex system displayed a slight decrease and convergence during the movement, indicating a conformational change in the complex. The RMSF of the system exhibited high values at the protein ends and low values in the middle, consistent with the typical pattern of stable binding systems. The free energy landscape showed two very close energy clusters, suggesting the complex may be somewhat less stable, but with a small difference between the two lowest energy conformations (Figure 6D).

## 4 Discussion

This study employed a cross‐sectional analysis using the NHANES database to further investigate the association between flavonoid intake and the prevalence of CKD. Although the renoprotective function of flavonoids has been demonstrated, this study was the first to identify the specific subclass and compound that were most strongly related to CKD prevalence. In this study, the NHANES and mediation analyses provided the foundational epidemiological evidence that allowed us to prioritize luteolin from among the flavonoids. Based on the robust and significant association between luteolin and reduced CKD risk observed in the population data, we subsequently focused on elucidating the underlying molecular mechanisms of luteolin. The network pharmacology and bioinformatics analyses provided a list of candidate targets (e.g., TP53, HSP90AA1, IL6, ESR1). Rather than presenting these targets in parallel, we used MR analysis as a “causal filter” to refine target selection. The MR analysis identified HSP90AA1 as a risk gene with a potential causal relationship with CKD, which allowed us to prioritize HSP90AA1 over the other candidates for further validation. We finally employed molecular docking techniques to validate whether luteolin could stably bind to this target, thereby providing support for the “luteolin‐HSP90AA1‐CKD” pathway at the structural biology level.

In this population‐based study, flavones—including apigenin and luteolin—showed the strongest inverse associations with CKD prevalence. Total flavones, apigenin, and luteolin intakes were all negatively related to CKD risk. Both the total intake of flavones and the intake of apigenin demonstrated linear correlations with CKD risk. Additionally, we observed a nonlinear correlation between the luteolin intake and CKD risk, with the optimal daily intake identified as 1.36 mg. Although both apigenin and luteolin have been demonstrated to have renal protective effects in animal models (Alsawaf et al. 2022), this study indicated a quantitatively greater association for luteolin than for apigenin.

Luteolin—abundant in tea (black and green), vegetables (onion, celery), fruits (apple, grape), and wine—has been associated with improved renal phenotypes in experimental models (Somerset and Johannot 2008). Luteolin attenuated renal damage through anti‐inflammatory, antiapoptotic, and antioxidant effects in the kidney ischemia–reperfusion (I/R) injury model (Wei et al. 2025). Additionally, luteolin has been found to reduce anemia caused by renal fibrosis through the SIRT1/FOXO3 pathway (Li et al. 2022). In diabetic animal models, luteolin has also been shown to decrease the deterioration of renal function (Xiong et al. 2020). However, clinical studies regarding the link between luteolin and the risk of CKD are still lacking. We therefore analyzed whether common causes and associated complications of CKD mediate the luteolin—CKD association. We found that blood pressure, blood glucose, blood lipids, and renal function indicators (creatinine, BUN, and urinary albumin‐to‐creatinine ratio, uACR) could not act as mediating factors. Whereas systemic inflammation, serum uric acid, albumin, and bicarbonate each accounted for 4%–8% of the total association. Experimental studies reported that luteolin could regulate inflammation via NFκB, MAPK, and AKT signaling pathways (Deng et al. 2025; Lu et al. 2024). Additionally, luteolin can facilitate the excretion of uric acid by modulating the expression of uric acid transporters (Yu et al. 2024). However, there is currently no evidence demonstrating luteolin's ability to regulate levels of bicarbonate and albumin, suggesting that further investigation into the mechanisms of action of luteolin is required.

KEGG enrichment analysis indicated that luteolin‐associated genes are over‐represented in pathways governing apoptosis, cellular senescence, and cell‐cycle control, as well as in HIF‐1, PI3K‐AKT, and TNF signaling and in the metabolism of arachidonic acid, nitrogen, and folate. Interleukin‐17, C‐type lectin receptor, and T‐cell receptor signaling cascades were also implicated. Four hub genes—TP53, IL6, ESR1, and HSP90AA1—were centrally positioned within these networks. Gene set enrichment and immune‐infiltration analyses further linked luteolin exposure to altered immune responses and differential leucocyte recruitment in CKD tissue. MR analysis identified HSP90AA1 as a putative CKD‐related target, and both molecular docking and molecular dynamics simulations indicated stable binding of luteolin to the HSP90AA1 ATP‐binding pocket.

HSP90AA1 is an ATP‐dependent molecular chaperone that orchestrates the folding and stabilization of client proteins involved in apoptosis, pyroptosis, ferroptosis, cell‐cycle progression, and signal transduction (Liu et al. 2025; Hoter et al. 2018). Its N‐terminal adenine‐binding pocket regulates ATPase activity and, consequently, chaperone function. In this study, luteolin, in the conformation corresponding to the lowest free energy, formed conventional hydrogen bonds and C–H bonds with Glu47, which is a key site reported to be involved in ATP hydrolysis of HSP90AA1 (Obermann et al. 1998). Furthermore, Ser50, which formed three C–H bonds with luteolin in the conformation corresponding to the lowest free energy in our study, together with Glu47, constitutes the previously reported Cdc37 binding site, while the inhibitor C‐316‐1 that binds to this site had been shown to exert a protective effect in cisplatin‐induced acute kidney injury (Liu, Liu, et al. 2022). Therefore, evidence from molecular dynamics simulations suggests that luteolin may act as a natural inhibitor of HSP90AA1, thereby preventing the maintenance of the conformation of proteins that play a key role in causing damage in CKD. Across disease models, elevated HSP90AA1 expression has been correlated with cancer, neurodegeneration, inflammation and viral infection, although its relevance to human CKD remains understudied (Yuan et al. 2022; Luo et al. 2010; Geller et al. 2011; Whitesell and Lindquist 2005). A 2025 study showed that perillaldehyde inhibited the protective effects of HSP90AA1 on ferroptosis‐related and pyroptosis‐related proteins, thereby attenuating sepsis‐induced acute kidney injury (Liu et al. 2025). Another study showed that the small molecule compound LM49 inhibited inflammatory responses and kidney fibrosis by binding to HSP90AA and blocking its interaction with HMGB1 (Wei et al. 2024). Therefore, luteolin may exert diverse biological functions by binding to HSP90AA1 and modulating its co‐chaperone proteins.

This study also has limitations. Firstly, the 24‐h dietary recall method used in NHANES, although a rigorously validated tool, primarily reflects short‐term intake. The mean flavonoid intake over 2 days used in this study might not fully capture day‐to‐day variability in an individual's diet or long‐term trends. Using such short‐term measurements to infer long‐term habitual intake relevant to chronic diseases may lead to misclassification of the true association. We propose that in future research, validated food frequency questionnaires (FFQs) or prospective cohorts with multiple 24‐h recalls should be utilized to comprehensively evaluate the association between long‐term dietary flavonoid intake and the risk of CKD. Secondly, the findings of this study are primarily applicable to the adult population in the United States. Due to differences in dietary patterns and risk factors for CKD across different countries and cultural backgrounds, extrapolating our results directly to other racial, ethnic, or geographical populations should be done with extreme caution. Thirdly, flavonoid intake data were obtained through dietary recall surveys, which could introduce recall bias. Furthermore, the specific mechanism through which HSP90AA1 affects CKD requires further investigation. Lastly, this research was conducted using cross‐sectional data, which limited its ability to establish causation and only allowed for the identification of associations. Due to the cross‐sectional nature of the data, our study could not rule out the possibility of reverse causality. Specifically, it is possible that early symptoms of CKD (such as decreased appetite, changes in taste, or dietary restrictions recommended by physicians) could lead to changes in patients' dietary habits, resulting in reduced luteolin intake, rather than low luteolin intake causing CKD. We conclude that confirming a causal relationship between luteolin intake and the incidence of CKD requires reliance on prospective cohort studies or randomized controlled trials.

## 5 Conclusions

In summary, luteolin exhibited the strongest inverse association with CKD prevalence among dietary flavonoids. Mediation analysis indicated that lower systemic inflammation, reduced serum uric acid, and higher bicarbonate and albumin levels collectively account for part of this relationship. Network pharmacology highlighted TP53, IL6, HSP90AA1, and ESR1 as central nodes within the luteolin‐associated interactome. MR analysis supported a genetically predicted link between HSP90AA1 and CKD risk, while molecular docking techniques indicated stable binding of luteolin to the HSP90AA1 N‐terminal pocket. Collectively, these findings implicate luteolin–HSP90AA1 interactions in the observed flavonoid–CKD association and warrant further mechanistic investigation.

## Author Contributions

Conception and design of the work: M.L., K.H., J.C., X.L., C.X., and Z.M.; acquisition, analysis, and interpretation of data: M.L. and K.H.; drafting: M.L., K.H., J.C., and X.L.; revising, M.L., K.H., C.X., and Z.M. All authors have read and agreed to the published version of the manuscript.

## Ethics Statement

The authors have nothing to report.

## Consent

The authors have nothing to report.

## Conflicts of Interest

The authors declare no conflicts of interest.

## Supporting information


**Table S1:** Results from MR‐Egger, weighted median, weighted mode, and simple mode.
**Table S2:** Results from heterogeneity tests.
**Table S3:** Results from horizontal pleiotropy tests.
**Table S4:** SNPs used as genetic tools for MR analysis.

## Data Availability

NHANES: https://www.cdc.gov/nchs/nhanes/index.htm; GWAS summary statistics: https://gwas.mrcieu.ac.uk/. GEO dataset GSE66494: https://www.ncbi.nlm.nih.gov/geo/query/acc.cgi?acc=GSE66494.
