# Supplementary material for: Renoprotective Role and Mechanisms of Luteolin in Chronic Kidney Disease: Insights From NHANES Data, Network Pharmacology, Mendelian Randomization, and Molecular Docking Techniques
Source: Food Sci Nutr. 2025 Nov 30;13(12):e71236. doi: 10.1002/fsn3.71236 (PMC12665478; doi:10.1002/fsn3.71236)
Supplement: Supplementary file 1 — Table S1: Results from MR‐Egger, weighted median, weighted mode, and simple mode. Table S2: Results from heterogeneity tests. Table S3: Results from horizontal pleiotropy tests. Table S4: SNPs used as genetic tools for MR analysis. [file FSN3-13-e71236-s001.docx]

Supplementary Methods

GWAS IDs for hub genes:

TP53: eqtl-a-ENSG00000141510

ESR1:eqtl-a-ENSG00000091831

IL6:eqtl-a-ENSG00000136244

HSP90AA1:eqtl-a-ENSG00000080824

Molecular dynamics simulations:

Amber99sb was chosen as the protein force field, Gaff2 was chosen as the ligand force field, the SPC/E water model was chosen to add solvents to the protein-ligand system and to create a water box with a periodic boundary of 1.2 nm. The Particle Mesh (PME) method was used to compute the long-range electrostatic interactions, and Monte Carlo Ion Placement method was used to introduce the appropriate amount of sodium and chloride ions to neutralize the charge of the whole system. Prior to formal simulation, system energy minimization and equilibra-tion were performed. After the system energy is minimized and equilibrated, molecular dynamics simulations are performed without any constraints for 100 ns at a time step of 2 fs.

Supplementary Materials 1

TP53 TNF NPC1 CFTR IL6 APP IL10 TERT STAT1 IFNG PRSS1 SNCA MET TTR MAPT AKT1 MTOR HMOX1 EGFR CBS MME MMP1 AR B2M MPO MMP9 F2 PTPN22 ABCB1 IL4 IL2 VEGFA CCND1 PON1 CXCR4 CD40LG ICAM1 PPARG AVPR2 ERBB2 ESR1 PYGL HLA-A SERPINE1 ZAP70 SLC2A1 INSR MT-CYB HIF1A PIK3CG SRC MMP2 PIK3R1 XDH KDR SLC6A3 CTSD MMP3 CASP3 PTGS2 FLT3 NFKBIA MIF RELA LDHA TBK1 GSTP1 PLG ACHE IL5 HMGCR GUSB ALK ABCC2 NR3C2 JUN CA2 SYK RB1 TYR MDM2 IGF1R CDK4 STAT5A GSK3B CYP3A4 MAOA ARG1 HSD17B10 PARP1 HDAC8 RAD51 PRKACA MAPK3 DPP4 PCNA CYP1B1 BACE1 REST ALOX5 GPI ABCG2 CYP19A1 CD38 CDK6 NTRK3 LGALS3 PRKDC CREB1 FOS NOX4 MMP12 MMP13 CYP1A1 CDK5 GLS CHUK CALM1 ACOX1 HSP90AA1 AHR UNC45A BCL2L1 PTGS1 NT5E KDM1A CASP9 AXL MAOB BIRC5 MCL1 GLRA1 KCND3 QDPR DYRK1A F13A1 CYP1A2 ADRB1 PGF ETS1 PDE11A ESR2 PTK2 CDK2 ABCC1 IGHG1 HDAC2 ANPEP ERAP1 AKR1B1 CYP2C8 HAO1 ADORA1 EIF4E PIM1 DRD4 F2R ACACA CAPN1 PMP2 RPS6KA3 SLC2A4 ALPI CDK1 PLK1 FLI1 CACNA1B CHKA ADORA2A CA4 LDHB VPS4A CHRNA4 TDP1 SLC6A5 CSNK2A1 CXCR1 APEX1 ODC1 ANTXR2 NR1I2 CA9 FASN SYN1 P4HB CASP7 LPO CDC25C DPP9 CCR4 E2F1 ACP1 EGLN1 CSNK2B GPBAR1 ELAVL1 ADK GPR35 ERN1 AKR1A1 TOP1 DAPK1 TOP2A HPGD PIN1 FCGRT GLO1 UQCRC1 CCNB1 TFPI CAMK2B PRKCZ CDK5R1 GSK3A GRIA2 UPF3B ALOX15 AURKB THRA CA12 AKR1C2 GSTO1 ALOX12 NEK2 CA5A CBR1 RCOR1 ST3GAL3 POLB OPRD1 P2RX4 AKR1C3 DPP7 HOXB13

Supplementary Tables

Supplementary Table 1. Results from MR-Egger, weighted median, weighted mode and simple mode.

| Gene | nsnp | Method | P-value | OR (95%CI) |
| --- | --- | --- | --- | --- |
| TP53 | 6 | MR Egger | 0.09025 | 1.59 (1.06 to 2.4) |
|  | 6 | Weighted median | 0.29895 | 1.15 (0.88 to 1.51) |
|  | 6 | Simple mode | 0.48834 | 1.17 (0.78 to 1.74) |
|  | 6 | Weighted mode | 0.34866 | 1.19 (0.85 to 1.66) |
| IL6 | 9 | MR Egger | 0.34048 | 1.51 (0.68 to 3.36) |
|  | 9 | Weighted median | 0.04618 | 1.3 (1 to 1.67) |
|  | 9 | Simple mode | 0.31092 | 1.28 (0.82 to 1.99) |
|  | 9 | Weighted mode | 0.20347 | 1.31 (0.89 to 1.92) |
| ESR1 | 8 | MR Egger | 0.52669 | 1.19 (0.71 to 1.99) |
|  | 8 | Weighted median | 0.16402 | 1.17 (0.94 to 1.46) |
|  | 8 | Simple mode | 0.48623 | 1.15 (0.8 to 1.65) |
|  | 8 | Weighted mode | 0.30058 | 1.16 (0.9 to 1.49) |
| HSP90AA1 | 44 | MR Egger | 0.00969 | 1.18 (1.05 to 1.33) |
|  | 44 | Weighted median | 0.00015 | 1.16 (1.08 to 1.26) |
|  | 44 | Simple mode | 0.01872 | 1.18 (1.03 to 1.34) |
|  | 44 | Weighted mode | 0.00105 | 1.18 (1.08 to 1.29) |

Supplementary Table 2. Results from heterogeneity tests.

| Gene | Method | Q | Q_df | Q_pval |
| --- | --- | --- | --- | --- |
| TP53 | MR Egger | 1.05730149111849 | 4 | 0.90098626571035 |
|  | Inverse variance weighted | 6.95203516843127 | 5 | 0.224232025772558 |
| IL6 | MR Egger | 11.4984833505724 | 7 | 0.118305102649712 |
|  | Inverse variance weighted | 11.9642968679929 | 8 | 0.152803992081773 |
| ESR1 | MR Egger | 4.65463130507393 | 6 | 0.588808361050076 |
|  | Inverse variance weighted | 4.65546358806992 | 7 | 0.701924959002761 |
| HSP90AA1 | MR Egger | 20.7053160291451 | 42 | 0.997626163821775 |
|  | Inverse variance weighted | 21.1346259711335 | 43 | 0.997947959872101 |

Supplementary Table 3. Results from horizontal pleiotropy tests.

| Gene | Egger_intercept | SE | P-value |
| --- | --- | --- | --- |
| TP53 | -0.08429276 | 0.034718278131865 | 0.0721438454327967 |
| IL6 | -0.024778327 | 0.0465304433919971 | 0.610845336385397 |
| ESR1 | -0.000977579 | 0.033885681187243 | 0.977920416226115 |
| HSP90AA1 | -0.007638449 | 0.0116578834337837 | 0.515900642068859 |

Supplementary Table 4. SNPs used as genetic tools for MR analysis.

| Gene | SNP ID | Effect Allele | Other Allele | Beta | SE | P-value | F-statistic |
| --- | --- | --- | --- | --- | --- | --- | --- |
| HSP90AA1 | rs1004783 | A | G | 0.183242 | 0.0128637 | 4.81504e-46 | 195.98847 |
|  | rs10140382 | T | C | 0.19165 | 0.0144515 | 3.86545e-40 | 232.99533 |
|  | rs10145749 | T | C | -0.114138 | 0.0167897 | 1.05925e-11 | 42.85564 |
|  | rs11160668 | A | G | 0.140947 | 0.0118556 | 1.35675e-32 | 135.2359 |
|  | rs111810795 | C | T | -0.15266 | 0.0209906 | 3.52128e-13 | 48.53516 |
|  | rs113400456 | A | G | -0.304006 | 0.0453071 | 1.94626e-11 | 56.52003 |
|  | rs116948773 | T | C | -0.168933 | 0.0265822 | 2.08161e-10 | 39.27228 |
|  | rs1190606 | G | A | -0.141743 | 0.0200963 | 1.74582e-12 | 50.47117 |
|  | rs1212692 | T | C | -0.264446 | 0.0225438 | 8.9043e-32 | 140.17365 |
|  | rs12435118 | C | T | 0.12266 | 0.0122757 | 1.65196e-23 | 107.68527 |
|  | rs12878795 | C | G | -0.329047 | 0.0275868 | 8.47813e-33 | 143.2125 |
|  | rs12880868 | A | G | 0.13899 | 0.021253 | 6.16027e-11 | 45.28538 |
|  | rs12884420 | G | A | 0.101855 | 0.0131143 | 8.05008e-15 | 57.96093 |
|  | rs12894354 | C | T | -0.0786084 | 0.0123236 | 1.7871e-10 | 43.09618 |
|  | rs140084989 | T | C | -0.249086 | 0.0335239 | 1.08418e-13 | 55.88669 |
|  | rs140810349 | A | G | -0.174976 | 0.0319801 | 4.45985e-08 | 29.71749 |
|  | rs142257199 | T | C | -0.319886 | 0.0251548 | 4.7698e-37 | 146.78962 |
|  | rs145916482 | A | G | -0.308887 | 0.0482108 | 1.48221e-10 | 55.41725 |
|  | rs148403098 | T | C | -0.303797 | 0.0279523 | 1.63042e-27 | 114.67352 |
|  | rs1678033 | A | G | -0.292944 | 0.0148424 | 1.03753e-86 | 356.1151 |
|  | rs17511942 | C | T | -0.278607 | 0.0299268 | 1.28056e-20 | 93.84874 |
|  | rs17512327 | G | A | -0.38135 | 0.0492008 | 9.11801e-15 | 81.51458 |
|  | rs188120581 | T | C | -0.239032 | 0.0394599 | 1.38e-09 | 54.52217 |
|  | rs188578402 | T | C | -0.281343 | 0.027014 | 2.12227e-25 | 116.64403 |
|  | rs2009575 | T | C | 0.0740916 | 0.0126998 | 5.4114e-09 | 32.34162 |
|  | rs2403017 | A | G | 0.385583 | 0.0153563 | 3.94457e-139 | 645.45123 |
|  | rs2476522 | C | T | -0.093478 | 0.0147986 | 2.6703e-10 | 36.08635 |
|  | rs2749908 | A | G | -0.137437 | 0.0141221 | 2.19786e-22 | 85.66464 |
|  | rs2896439 | A | G | 0.0732025 | 0.0124672 | 4.31609e-09 | 32.99792 |
|  | rs35471611 | C | T | -0.162605 | 0.0233853 | 3.56615e-12 | 48.66526 |
|  | rs3742421 | G | C | -0.127713 | 0.0194011 | 4.60999e-11 | 40.41296 |
|  | rs45511198 | C | A | -0.180667 | 0.0196228 | 3.35351e-20 | 77.79168 |
|  | rs4601978 | T | C | -0.251316 | 0.0143293 | 7.26942e-69 | 278.19907 |
|  | rs516805 | T | G | 0.084601 | 0.0129217 | 5.86273e-11 | 38.90974 |
|  | rs60184724 | C | T | -0.184545 | 0.0212202 | 3.4135e-18 | 68.65403 |
|  | rs62006410 | T | C | -0.0901535 | 0.0148746 | 1.35279e-09 | 38.24934 |
|  | rs6575895 | T | C | 0.126145 | 0.0184677 | 8.44501e-12 | 42.79736 |
|  | rs7140813 | T | C | -0.25759 | 0.01413 | 2.97235e-74 | 304.93095 |
|  | rs7156529 | A | G | -0.373891 | 0.02994 | 8.6816e-36 | 142.5432 |
|  | rs7159203 | T | C | -0.128002 | 0.0234286 | 4.66573e-08 | 27.09489 |
|  | rs75448782 | T | G | -0.339679 | 0.0341176 | 2.36974e-23 | 90.591 |
|  | rs75636299 | A | C | -0.331567 | 0.0247309 | 5.50174e-41 | 163.19462 |
|  | rs77011345 | C | T | -0.416603 | 0.0459456 | 1.21899e-19 | 101.7078 |
|  | rs8007105 | A | C | -0.217435 | 0.0200617 | 2.26412e-27 | 113.16407 |
| ESR1 | rs11752282 | A | G | 0.10894 | 0.0195506 | 2.51542e-08 | 35.46207 |
|  | rs11757393 | A | G | 0.125909 | 0.0184528 | 8.9043e-12 | 44.65234 |
|  | rs117819917 | T | C | -0.245299 | 0.0367116 | 2.35722e-11 | 47.25658 |
|  | rs145647131 | A | G | -0.239405 | 0.0405085 | 3.41688e-09 | 36.10039 |
|  | rs2982565 | A | G | -0.141257 | 0.0151484 | 1.10969e-20 | 84.02368 |
|  | rs3020333 | G | A | -0.169439 | 0.0118573 | 2.52639e-46 | 194.8798 |
|  | rs852002 | C | T | 0.0784752 | 0.0119066 | 4.37421e-11 | 41.64041 |
|  | rs977533 | A | G | -0.0852146 | 0.0120494 | 1.52721e-12 | 47.93912 |
| IL6 | rs10250252 | C | T | -0.0966175 | 0.0145491 | 3.1153e-11 | 42.43191 |
|  | rs10265117 | A | G | -0.172352 | 0.0181279 | 1.9476e-21 | 87.30064 |
|  | rs111372030 | A | G | 0.151606 | 0.0220262 | 5.86813e-12 | 52.20904 |
|  | rs117122223 | G | T | -0.104469 | 0.0151567 | 5.47772e-12 | 46.90308 |
|  | rs11766273 | A | G | 0.140597 | 0.0218176 | 1.16209e-10 | 41.03465 |
|  | rs11766947 | C | T | 0.111166 | 0.0133017 | 6.42244e-17 | 68.19748 |
|  | rs4621699 | G | A | -0.0832732 | 0.0141648 | 4.12753e-09 | 34.12129 |
|  | rs71520366 | C | T | 0.0670306 | 0.0119367 | 1.9611e-08 | 30.79657 |
|  | rs724081 | T | C | -0.152745 | 0.0271591 | 1.86372e-08 | 33.94987 |
| TP53 | rs138420351 | T | C | -0.282676 | 0.0431929 | 5.96486e-11 | 49.45804 |
|  | rs143094271 | A | G | -0.290607 | 0.0377259 | 1.32709e-14 | 61.7707 |
|  | rs1641518 | G | A | 0.105475 | 0.0175248 | 1.75711e-09 | 34.07959 |
|  | rs35850753 | T | C | -0.356015 | 0.0377475 | 4.03924e-21 | 92.603 |
|  | rs57985740 | G | A | 0.080471 | 0.0147073 | 4.46488e-08 | 28.77235 |
|  | rs6503048 | T | C | -0.131582 | 0.022775 | 7.5774e-09 | 31.74235 |
